# Changing Trends in School Absenteeism and Identification of Associated Factors in Adolescents with Atopic Dermatitis

**DOI:** 10.3390/children10121918

**Published:** 2023-12-12

**Authors:** Dong Wan Kang, Sung Hoon Kim, Yechan Kyung, Hae Jeong Lee

**Affiliations:** Department of Pediatrics, Samsung Changwon Hospital, Sungkyunkwan University School of Medicine, Changwon-si 51353, Republic of Korea; eugenestyle.kang@samsung.com (D.W.K.); hee7307@skku.edu (S.H.K.); drchany@skku.edu (Y.K.)

**Keywords:** adolescent, atopic dermatitis, depression, Korean youth risk behavior web-based survey, school absenteeism

## Abstract

Atopic dermatitis (AD) has a negative influence on school attendance. We aimed to identify factors associated with school absenteeism in adolescents with AD. We used data from the 3rd to 11th annual Korean Youth Risk Behavior Web-based Survey completed from 2007 to 2015. Survey data were obtained from a stratified, multistage, clustered sample. Participants responded to the question “have you ever been diagnosed with AD?” Factors associated with AD-related school absenteeism (ADSA), which is defined as at least one school absence due to AD, were evaluated. Among the 141,899 subjects, the prevalence of AD increased (17.3% to 24.2%), while that of ADSA decreased (7.3% to 2.6%) from 2007 to 2015. Compared to adolescents without ADSA, those with ADSA were more likely to be male, middle school students, and have negative mental health states, including suicidality. In the multivariate logistic regression model, the association of sleep dissatisfaction and depression with ADSA was high (adjusted odds ratio, 6.12; 95% confidence intervals, 4.61–7.95; and 5.44; 5.23–5.67, respectively). The prevalence of ADSA has decreased despite an increase in the prevalence of AD in Korean adolescents; however, it is important for pediatricians to screen for factors associated with ADSA to improve school attendance in adolescents with AD.

## 1. Introduction

Atopic dermatitis (AD) is a chronically relapsing skin disease present in up to 20% of children and up to 3% of adults [1,2]. Marked by severe itching, AD is a chronic inflammatory skin disease prevalent in early childhood, often diminishing with age. However, its persistence into adolescence or late onset is linked to higher severity [3]. The etiology and pathogenesis of AD are not fully understood, but current understanding suggests a combination of impaired skin barrier function, immune dysregulation, and dysbiosis of the skin or gut microbiome [4]. The clinical diagnosis of AD involves assessing pruritus, characteristic skin lesions, a chronic clinical course, and personal or family history of allergic diseases, with differential diagnosis considering conditions such as contact dermatitis, seborrheic dermatitis, and psoriasis [5,6]. Treatment for atopic dermatitis includes skincare involving cleaning and moisturizing, environmental control, and medical therapy using topical corticosteroids or immunosuppressants, with recent advancements focusing on individualized approaches based on distinct phenotypes and endotypes [6]. In addition to the high prevalence and deleterious clinical effects, subjects with AD are most likely to suffer considerable poor attendance or performance at work or school related to its negative impact on children’s sleep and behavior [2,7,8,9,10,11,12]. In the previous study, 32% of participants believed that AD affected their school or work life and reported an average of 2.5 days of absenteeism from schoolwork because of an AD flare [13]. In the United States (US), adults with AD were significantly more likely to have lost ≥6 workdays compared to those without AD (odds ratio [OR] 1.53, 95% confidence interval [CI] 1.26–1.84) [14]. In Canada, 20% of children with AD missed school related to their AD symptoms, and of those respondents, 23% had ≥10 missing school days in the past year [15]. Additionally, 1.0% of 20,094 adolescents with AD were absent from school for ≥7 days in the past year in Korea [16].

AD-related school absenteeism can result from a combination of the underlying degree of their disease’s severity, an acute AD flare, sleep impairment due to itching, poor AD control, longer duration of AD, low self-esteem due to AD visibility, restriction of participation in leisure and sports, the psychological stress associated with a chronic illness, comorbid asthma or allergic rhinitis, and routine health care visits [7,8,12,13,15,16,17]. In the US, from the perspective of racial and ethnic differences, non-Hispanic black and Hispanic children demonstrated 1.5- and 3.4-fold higher likelihoods of experiencing school absenteeism due to AD, respectively, compared to non-Hispanic white children [17].

Among the general US adolescent population, 16% of students were chronically absent (defined as missing at least 15 days of the school year) [18]. Students with chronic absenteeism face an increased risk of academic difficulties and a higher likelihood of dropping out [19,20]. However, school absenteeism is not only a relevant issue in the normal process of schooling [21] but is also associated with lifelong negative social and health sequelae, such as dangerous sexual behavior, teenage pregnancy, psychiatric disorders, and tobacco, alcohol, and other substance abuse [19,20,21,22]. Although previous research have examined the association between school absenteeism of general adolescents and overlapping individual, medical, family, and social factors, and the impact of AD on school absence [2,7,8,9,12,13,15], to the best of our knowledge, nationally representative estimates of associations between AD-related school absenteeism and associated factors, especially in the adolescents, are not well evaluated. If these associated factors were better understood, this information could be crucial to not only educators but also to clinicians considering school-based efforts to improve AD control.

Given this background, we hypothesized that among adolescents with AD, subjects with school absenteeism are prone to engaging in problematic behaviors, such as skipping breakfast, consuming alcohol, and smoking, and have more negative mental health statuses, such as experiencing more stress, dissatisfaction with sleep, depression, and suicidality, than individuals that were not absent from school. As such, the objectives of this study are to identify the changing trends in the proportion of AD-related school absenteeism in Korean adolescents, to compare the differences according to school absenteeism among adolescents with AD, and then to identify the associated factors for these school absences, which is the main purpose of our study.

## 2. Methods

### 2.1. Study Design and Study Population

This study performed a secondary analysis utilizing data obtained from the 3rd through 11th Korean Youth Risk Behavior Web-based Surveys (KYRBSs), conducted over 9 years from 2007 to 2015 [23,24]. The KYRBS initiative was established in 2002 as a collaborative effort of the Korean Ministry of Education (MOE), Ministry of Health and Welfare, and the Centers for Disease Control and Prevention (KCDC) [23,24]. To qualify the surveys, the understanding, reliability, and validity of each question were examined every year by the KCDC. The reliability estimates for the KYRBSs questionnaire have been rated as having good validity [23]. 

The KYRBS is an annual cross-sectional online survey designed to evaluated various health-risk behaviors among Korean adolescents aged 12–18 years, covering students from the first year of middle school to the third year of high school in the Korean education system. The Institutional Review Board (IRB) of the KCDC approved this research for every year of the survey. The KYRBS utilized a three-stage stratified random cluster sampling method to achieve a nationally representative sample. In the stratification stage, the participants were stratified according to geographic region and school type (public or private, coeducation, and vocational school) to minimize sampling errors. In the sample allocation, approximately 75,000 students from 400 middle schools and 400 high schools were selected by proportional sampling to match the study population. In the stratified cluster sampling, one class from each grade was selected using stratified cluster extraction from the selected schools. 

Written informed consent from parents or legal guardians was submitted before participating in the survey. Survey participation excluded age-eligible respondents with absenteeism, including dropout or expulsion, those with special needs (such as development disabilities), and those with dyslexia. Students voluntarily participated at their schools’ computer laboratories using their randomly assigned and unique identification numbers. After logging on to the website of KYRBS, participants had to answer each question; nonresponses were not accepted. However, certain data were considered missing due to logical errors or were considered outliers. The reasons for nonparticipation were not specified; however, adolescents had the option to withdraw or choose not to complete the surveys or discuss the assent process. Of the 685,710 targeted adolescents, 660,607 were included in the analysis. 

### 2.2. Evaluation Indices

#### 2.2.1. Atopic Dermatitis and School Absenteeism

Lifelong diagnosis of AD was assessed by answering “Yes” to the following question: “Have you ever been diagnosed with AD by a doctor at any stage in your life?” The severity of current symptoms and modality of treatment were not evaluated. Our primary outcome of interest was school absenteeism due to AD (hereafter referred to as “AD-related school absenteeism [ADSA]”, which was defined as being absent for at least one day in our study). Participants were asked the following the question: “Within the past 12 months, about how many days of school did you miss due to your AD?” Their responses were classified into four categories for our study: no absences, 1–3 days, 4–6 days, and ≥7 days. 

#### 2.2.2. Demographic and Socioeconomic Characteristics

Information on sex, school grade, perceived socioeconomic status (SES), and academic achievements was assessed. Participants were categorized into middle school and high school. The degree of SES and academic achievement were evaluated by the following questions: “During the past 12 months, how would you subjectively rate your SES and academic performance, respectively?” Responses were categorized into high (high or middle–high), middle (middle), and low (middle–low or low). 

#### 2.2.3. Dietary, Smoking, and Drinking Behaviors

Breakfast consumption frequencies were investigated with the question, “In the past 7 days, how many days did you eat breakfast?” Participants could choose from the option “never, 1, 2, 3, 4, 5, 6, or 7 days”. Individuals who reported eating breakfast less than twice a week were classified as ‘skipped breakfast’ in accordance with the criteria established by the survey. Current smoking status at the time of the study was assessed by a reply of “more than 1 day over the past month” to the question “Do you smoke?” For alcohol consumption status, current drinkers were indicated by a reply of “More than 1 day” to the question “How many days did you drink at least one-shot glass of alcohol in the month preceding this survey?”

#### 2.2.4. Emotional States and Suicidality

Subjective health was assessed with the question, “How healthy do you usually feel?”, with response categories including healthy, average, and unhealthy. Perceived happiness was assessed by the question, “How happy do you usually feel?”, and their responses were classified as happy, average, or unhappy. Sleep satisfaction levels were assessed by the question, “How satisfied are you with your sleep during the last week?”, and the responses were categorized into three categories: enough, average, or not enough. Additionally, perceived stress status was also evaluated through the question, “To what degree are you usually stressed?”, and the replies were classified as follows: high, average, and low. Finally, depressive mood, suicide ideation, and suicide attempts were assessed by the following questions: “Within the last year, did you feel sad, blue, or depressed, resulting in a cessation of your usual activities almost every day for two weeks or longer?”; “Within the last year, have you ever seriously considered of committing suicide?”; and “Have you ever attempted suicide?” Binary responses (yes or no) were recorded. 

### 2.3. Statistical Analysis

All statistical analyses were conducted utilizing the complex sample procedures available in the Statistical Package for the Social Sciences (SPSS) software program version 21.0 (IBM Corp., Armonk, NY, USA). Given that the KYRBS data were gathered through a representative, stratified, and clustered sampling method, data were weighted to account for the sample design. The chi-squared test was used for the categorical variables and independent *t*-tests were used for the continuous variables in order to compare the general characteristics between adolescents with and without ADSA. To identify the factors associated with ADSA, we performed a multivariable logistic regression analysis with the addition of several variables. The results were expressed as adjusted ORs (aORs) and 95% confidence intervals (CIs). Statistical significance was indicated by *p* < 0.05 in all tests. 

## 3. Results

### 3.1. Changing Trends in the Rates of School Absenteeism among Adolescents with Atopic Dermatitis

**Figure 1:**
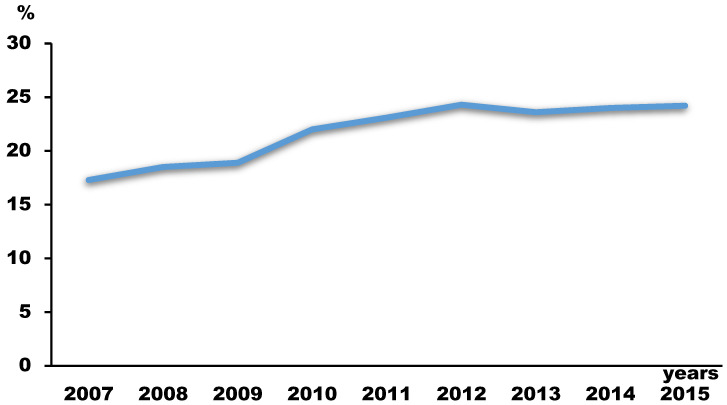
Changing trends in the prevalence of atopic dermatitis.

**Figure 2:**
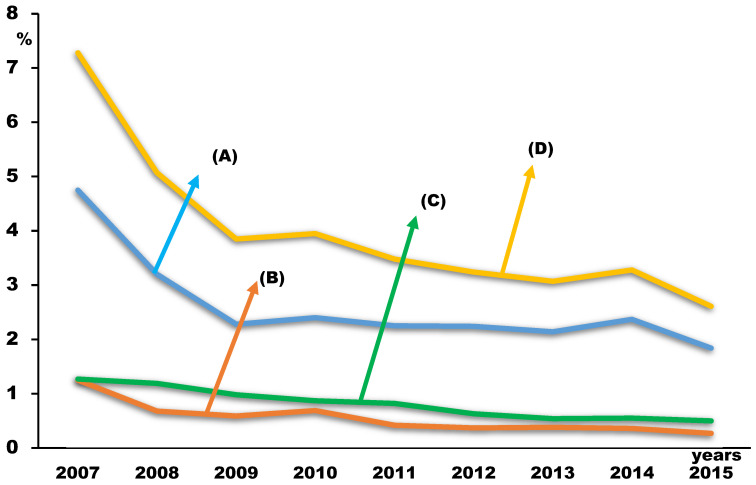
Changing trends in school absenteeism among adolescents with atopic dermatitis according to the days absent (1–3 days (A), 4–6 days (B), more than 7 days (C), and more than one day as total (D)).

During the 9 years, 660,607 subjects completed the survey, with a total response rate of 96.4% (range from 94.8% to 97.7%, Table 1). Among the subjects with AD (*n* = 141,899), there were 5468 (4.0%) subjects with ADSA (Table 2). While the prevalence of AD in adolescents increased (from 17.3% in 2007 to 24.2% in 2015, Table 1 and Figure 1), the prevalence of ADSA decreased during the survey years (7.3% in 2007, 2.6% in 2015, Table 1 and Figure 2). The prevalence rates of all three of the groups according to their durations of school absenteeism among participants with ADSA also decreased during the survey years (Table 1 and Figure 2).

### 3.2. Differences According to School Absenteeism among Adolescents with Atopic Dermatitis

Those with ADSA were more likely to be male, middle school-grade students, have lower SES, struggle with academics, frequently skip breakfast, often drink alcohol, and currently smoke. They also reported feeling less healthy and less happy, along with experiencing higher levels of stress, dissatisfaction with sleep, depression, thoughts of suicide, and actual suicide attempts compared to those without ADSA. (Table 2).

### 3.3. Differences According to the Duration of the School Absences among Adolescents with Atopic Dermatitis-Related School Absenteeism

A total of 65.7% of the students were absent for 1–3 days (1–3-day group), 20.5% were absent for more than 7 days (≥7 day group), and 13.8% were absent for 4–6 days (4–6 day group) (Table 3). The proportion of female students was highest in the 1–3-day group; however, the proportion of male students was highest among the 4–6-day group. The participants that missed more than seven days of school were more likely to have low SES and low academic achievements. They were also more likely to skip breakfast, frequently drink alcohol, be current smokers, and report more negative mental health variables except for depression (participants in the 4–6-day group had the highest proportion of depression). Additionally, they were more likely to engage in suicidal ideation and attempt suicide than those in the 1–3-day and 4–6-day groups.

### 3.4. Associated Factors for School Absenteeism in Individuals with Atopic Dermatitis 

After adjustment, both being male and attending middle school were associated with ADSA (Table 4). High and low SES, breakfast skipping, alcohol consumption, and smoking were also associated with ADSA. Among the mental health variables, sleep dissatisfaction was the most strongly associated (aOR: 6.12, 95% CI: 4.61–7.95), and then depression was the second most significantly associated (aOR: 5.44, 95% CI: 5.23–5.67). In addition, suicidal ideation and suicide attempt were also significantly associated (aOR 3.12, 95% CI: 1.93–4.13, aOR 2.30, [95% CI; 2.04–2.60], respectively). 

**Figure 3:**
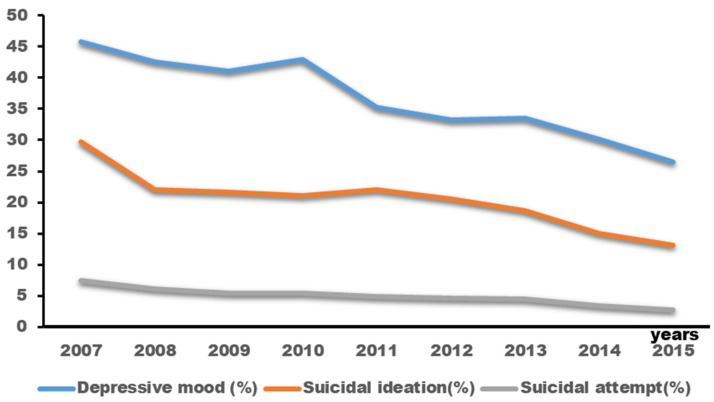
Changing trends in depressive mood, suicidal ideation, and suicide attempts among adolescents with atopic dermatitis during the survey years.

Figure 3 illustrates a decreasing trend in the prevalence rates of depressive mood, suicidal ideation, and suicide attempts among adolescents with atopic dermatitis throughout the survey (depressive mood: 45.8% in 2007 to 26.5% in 2015; suicidal ideation: 27.9% in 2007 to 13.1% in 2015; suicide attempts: 7.5% in 2007 to 2.7% in 2015).

## 4. Discussion

The main finding of this study was that adolescents with ADSA were more likely to engage in problematic behaviors and to report more negative mental health states, including suicidality, than those without ADSA, which was consistent with our hypothesis. Among several associated factors, sleep dissatisfaction and depression were most strongly associated with ADSA. Another notable finding of our study was that while the prevalence of AD increased, the prevalence of ADSA decreased during the survey years. Although the causal relationship—whether the severity of AD has become milder or treatment has been more effective during survey—cannot be distinctly explained due to the study design, this finding is likely to be interpretable in several ways. First, from the perspective of public education, the effect of the Wee class project can be considered. It was initiated in 2008 by the MOE to provide comprehensive support services of psychologists to students, including counseling for depression, suicidality, bullying, and low school performance [25]. Second, the manuals containing step-by-step response strategies for any school absenteeism (regardless of the type) were produced by the MOE and are being used in the field of education [26]. Regarding public health, we believe that the findings of decreasing prevalence of ADSA may be the result of efforts to improve the effectiveness of AD therapy. For example, since 2010, the local governments of Korea have established educational and informational centers for the preventing of allergic diseases through locally representative medical institutions [27]. Additionally, well-structured educational programs are being offered in numerous hospitals in Korea, potentially enhancing treatment compliance and ultimately improving treatment outcomes [27]. 

In meta-analyses, some of the risk factors of school absenteeism were related to the characteristics of the youth (e.g., age, substance abuse, internal and external problematic behaviors, and poor physical health), their family (e.g., parental psychiatric problems and unemployment, low SES, history of child abuse, and family breakup), their school (e.g., poor teacher quality), or their peer group (e.g., antisocial or delinquent peers) [19,20,21]. In particular, from the point of view that emotional disorders are identified as leading contributors to the burden of disease in adolescents [28,29], many studies have shown a relationship between emotional disorders and school absenteeism in the general adolescent population [28,29,30].

Similarly, our study identified several mental health variables as being significantly associated with ADSA. AD burdens adolescents in multiple ways [2,7,8,11,12]. Embarrassment due to disfigurement leads to reduced self-esteem, social stigmatization, and social isolation, thus causing stress in social relationships and bullying [8,31,32]. Bullying is another important and established cause of school absenteeism [18], and another of these is impairment of sleep. The children with AD are noted to have poor sleep efficiency and more daytime sleepiness due to frequent night wakening [12,33]. This exhaustion from sleep impairment leads to poor concentration at school [9,12,13]. These psychological stresses and AD symptoms, especially itching, form a vicious cycle; AD may be worsened by emotional stress, and patients with AD are more prone to express psychosomatic symptoms than normal controls [34]. Associations between depression and AD in adolescents are well known [31,34,35]. Emotional disorders, especially depression, in adolescents with AD may lead to school absenteeism through social withdrawal, loss of motivation, sleep disturbances, and low energy as in the general adolescent population [30].

In our study, those with ADSA were predominantly male adolescents, and being male was a significant factor associated with ADSA. This is similar to a finding of a previous study revealing that one of the main factors associated with school absenteeism is being male [36]. However, this is something to consider from the perspective of the association between sex predominance and AD. Sex is a biological variable that should be considered in immunological studies; it is well demonstrated that respiratory allergies, especially asthma, are prevalent in male students during childhood, while it is more frequent in female students from adolescence to adulthood [37]. However, no sex differences were found for exercise-induced anaphylaxis [37]. In terms of AD and food allergies, there were conflicting results, with male adolescents more commonly having insect venom allergies and female adolescents more commonly having drug allergies [37]. Although interventions to reduce school absenteeism should pay special attention to male adolescents with AD based on the results of our study, further well-controlled prospective studies are necessary to confirm these results.

Lastly, in the present study, although the statistical significance was relatively small, adolescents with ADSA were predominantly middle school students; following the logistic regression analysis, middle school enrollment was significantly associated with ADSA. Although it was not known whether the AD symptoms of middle school students were more severe than those of high school students in our study, these findings may be because middle school students are more vulnerable than high school students to psychosocial changes and lack the ability to control their emotional instability [28,29,30]. 

This study had some limitations. Many of its limitations are related to those associated with secondary analysis studies that are based on self-reported questionnaires. First, because this study was cross-sectional in design, causal relationships between associated factors and ADSA are not identifiable. In particular, the causes of the increasing prevalence of AD but decreasing proportions of ADSA could not be explained, as mentioned earlier, and the aforementioned well-known risk factors for school absenteeism could not be assessed [19,20]. Well-controlled prospective studies are necessary to overcome this limitation. Second, in the KYRBSs, school absenteeism was not addressed in adolescents without AD; therefore, we could not compare the differences in school absenteeism and associated factors among adolescents with and without AD, which is a major limitation. However, because it is well known that adolescents with AD are more likely to be absent from school compared to their healthy peers [2,7,8,11,12], we focused on which factors are involved in school absences of adolescents with AD. Third, the surveys used to collect data did not encompass adolescents who had either dropped out of school or were subject to expulsion. In addition, if the nonparticipants were more prone to having Atopic Dermatitis (AD), and the primary reasons for nonparticipation were related to AD conditions, the estimates could be somewhat biased. Fourth, we evaluated only the lifelong diagnosis of AD regardless of the subjects’ current symptoms; therefore, we could not separate currently active AD from previous (but treated or well-controlled) AD. We could not analyze presence, modality and failure of treatment, current symptoms, and severity because these data were not available from the survey. This led to the misclassification of some AD cases; if these data were available, some conclusions would probably change. Despite these limitations, this study had several strengths that improve on the findings of previous reports of school absenteeism in adolescents with AD. To the best of our knowledge, factors associated with ADSA in adolescents have not been explored with a large population dataset, as we did with this study. Also, this study was based on nationwide surveys with high response rates (96.4%). An equal proportion of socioeconomically diverse middle school and high school students were assessed annually, and all analyses in this study were based on sample weights. This can allow for the generalization of the results in Korea. Additionally, this study examined trends in ADSA over time and adopted a comprehensive approach to identify factors associated with ADSA.

## 5. Conclusions

In conclusion, our findings suggest that while the prevalence of AD in adolescents in Korea increased, the prevalence of ADSA decreased during the survey years, with a mean prevalence of 4.0%. However, because subjects with ADSA engage in more problematic behaviors and have more negative mental health statuses than those without ADSA, when assessing adolescents with AD, physicians should inquire about school absences; furthermore, screening procedures for associated factors related to ADSA should be emphasized in order to reduce ADSA. Mental health conditions that interfere with school attendance should be treated with a multidisciplinary team approach, including proper mental health referrals if necessary. Management of AD should include clear expectations about school attendance, and physicians, families, and schools should be key collaborators in interventions to reduce ADSA.

## Figures and Tables

**Table 1:** Changing trends in the prevalence of atopic dermatitis and school absenteeism among adolescents with atopic dermatitis in Korea from 2007 to 2015.

	Years								
	2007	2008	2009	2010	2011	2012	2013	2014	2015
Targeted participants (*n*)	78,834	79,099	76,937	74,980	79,202	76,980	75,149	74,167	70,362
Total participants (*n*)	74,698	75,238	75,066	73,238	75,643	74,186	72,435	72,060	68,043
Participant rate (%)	94.8	95.1	97.6	97.7	95.5	96.4	96.4	97.2	96.7
Participants with AD (*n*)	12,535	13,658	13,934	15,892	17,421	17,971	16,916	17,232	16,340
Prevalence of AD (%)	17.3	18.5	18.9	22.0	23.1	24.3	23.6	24.0	24.2
School absenteeism due to AD									
(1) ADSA (*n*, %)	912 (7.3)	693 (5.1)	536 (3.9)	628 (4.0)	607 (3.5)	582 (3.2)	519 (3.1)	565 (3.3)	426 (2.6)
(2) 1–3 days (*n*, %)	596 (4.8)	437 (3.2)	318 (2.3)	381 (2.4)	392 (2.3)	402 (2.3)	362 (2.1)	409 (2.4)	301 (1.8)
(3) 4–6 days (*n*, %)	157 (1.3)	93 (0.7)	82 (0.6)	109 (0.7)	73 (0.4)	66 (0.4)	65 (0.4)	62 (0.4)	44 (0.3)
(4) ≥7 days (*n*, %)	159 (1.3)	163 (1.2)	136 (1.0)	138 (0.9)	142 (0.8)	114 (0.6)	92 (0.5)	94 (0.6)	81 (0.5)

AD: atopic dermatitis; ADSA: atopic dermatitis related school absenteeism [ADSA], which was defined as absent school for at least one day in our study.

**Table 2:** Differences according to school absenteeism among 141,899 adolescents with atopic dermatitis.

	School Absenteeism		
	Absence (*n* = 136,431, 96.0%)	Presence (*n* = 5468, 4.0%)	*p*-Value
Sex			<0.001
Female	76,610 (55.02)	2286 (40.61)	
Male	59,821 (44.98)	3182 (59.39)	
Grade			0.029
Middle school	70,485 (50.68)	3040 (54.69)	
High school	65,946 (49.32)	2428 (45.31)	
Subjective economic state			<0.001
High	41,328 (31.27)	1747 (31.82)	
Middle	64,881 (47.07)	2025 (36.83)	
Low	30,222 (21.66)	1696 (31.35)	
Academic achievement			<0.001
weHigh	52,080 (38.19)	1543 (27.91)	
Middle	36,766 (26.98)	1230 (23.17)	
Low	47,585 (34.83)	2695 (48.92)	
Breakfast consumption			<0.001
<2 times/week	28,033 (20.63)	1732 (31.31)	
≥2 times/week	108,398 (79.37)	3736 (68.69)	
Current drinking			<0.001
No	110,378 (80.86)	3545 (63.56)	
Yes	26,053 (19.14)	1923 (36.44)	
Current smoking			<0.001
No	123,637 (90.47)	3999 (72.87)	
Yes	12,794 (9.53)	1469 (27.13)	
Subjective healthiness			<0.001
Healthy	85,421 (62.99)	3001 (54.21)	
Average	38,472 (27.91)	1597 (28.01)	
Unhealthy	12,538 (9.10)	870 (17.78)	
Subjective happiness			<0.001
Happy	76,272 (55.96)	2629 (48.01)	
Average	42,152 (30.96)	1731 (31.30)	
Unhappy	18,007 (13.09)	1108 (20.69)	
Perceived stress			<0.001
High	61,406 (44.85)	2863 (51.36)	
Average	55,216 (40.70)	1861(34.33)	
Low	19,809 (14.45)	744 (14.31)	
Sleep satisfaction			<0.001
Enough	53,281 (38.95)	1264 (22.77)	
Average	36,967 (27.12)	1010 (19.12)	
Not enough	46,183 (33.93)	3194 (58.11)	
Depression			<0.001
No	88,003 (64.58)	2562 (46.74)	
Yes	48,428 (35.42)	2906 (53.26)	
Suicidal ideation			<0.001
No	109,735 (80.45)	3669 (66.92)	
Yes	26,969 (19.55)	1799 (33.08)	
Suicide attempt			<0.001
No	130,300 (95.52)	4646 (84.35)	
Yes	6131 (4.48)	822 (15.65)	

Survey data were weighted to ensure statistical representation of the general population according to the sample design. The chi-squared test was employed to assess statistical differences among categorical data, while the independent *t*-test was utilized for continuous variables.

**Table 3:** Differences according to the duration of school absence among 5468 adolescents with atopic dermatitis-related school absenteeism.

	School Absenteeism (*n* = 5468)			
	1~3 Days (*n* = 3598, 65.7%)	4~6 Days (*n* = 751, 13.8%)	≥7 Days (*n* = 1119, 20.5%)	*p*-Value
Sex				<0.001
Female	1651 (44.69)	254 (31.91)	381 (34.11)	
Male	1947 (55.31)	497 (68.09)	738 (65.89)	
Grade				0.029
Middle school	2003 (53.29)	417 (53.54)	620 (52.90)	
High school	1595 (46.71)	334 (46.46)	499 (47.10)	
Subjective economic state				<0.001
High	1163 (32.59)	241 (32.00)	343 (32.46)	
Middle	1423 (39.71)	276 (37.80)	326 (28.12)	
Low	1012 (27.69)	234 (30.21)	450 (39.42)	
Academic achievement				<0.001
High	1027 (29.04)	197 (27.39)	319 (30.60)	
Middle	864 (23.78)	169 (22.72)	197 (16.62)	
Low	1707 (47.18)	385 (49.89)	603 (52.79)	
Breakfast consumption				<0.001
<2 times/week	1068 (29.42)	251 (32.61)	413 (38.86)	
≥2 times/week	2530 (70.58)	500 (67.39)	706 (61.14)	
Current drinking				<0.001
No	2452 (68.53)	449 (57.78)	644 (56.37)	
Yes	1146 (31.47)	302 (42.22)	475 (43.63)	
Current smoking				<0.001
No	2829 (78.61)	507 (65.94)	663 (56.89)	
Yes	769 (21.39)	244 (34.06)	456 (43.11)	
Subjective healthiness				<0.001
Healthy	1977 (54.66)	405 (54.24)	619 (54.21)	
Average	1102 (31.01)	226 (29.21)	269 (23.82)	
Unhealthy	519 (14.34)	120 (16.55)	231 (21.97)	
Subjective happiness				<0.001
Happy	1792 (49.92)	337 (44.50)	500 (44.01)	
Average	1149 (32.42)	263 (35.12)	319 (26.22)	
Unhappy	657 (17.66)	151 (20.68)	300 (29.77)	
Perceived stress				<0.001
High	1857 (51.64)	397 (52.34)	609 (56.76)	
Average	1287 (35.41)	251 (32.76)	323 (28.24)	
Low	454 (12.96)	103 (14.90)	187 (15.00)	
Sleep satisfaction				
Enough	1104 (30.48)	203 (27.01)	314 (27.64)	
Average	796 (22.91)	175 (23.51)	193 (17.27)	
Not enough	1698 (46.61)	373 (49.48)	612 (55.09)	
Depression				<0.001
No	1763 (47.82)	305 (41.86)	494 (43.11)	
Yes	1835 (52.18)	446 (58.14)	625 (56.89)	
Suicidal ideation				<0.001
No	2519 (70.21)	474 (61.04)	676 (59.87)	
Yes	1079 (29.79)	277 (38.96)	443 (40.13)	
Suicide attempt				<0.001
No	3201 (88.66)	599 (78.36)	846 (74.62)	
Yes	397 (11.34)	152 (21.64)	273 (25.38)	

Survey data underwent weighting to ensure statistical representation of the general population, aligning with the sample design. The chi-squared test was employed to assess statistical disparities among categorical data, while the independent *t*-test was utilized for continuous variables.

**Table 4:** Associated factors for school absenteeism among adolescents with atopic dermatitis.

	Crude		Adjusted	
	OR (95% CI)	*p*-Value	OR (95% CI)	*p*-Value
Sex				
Female	Reference		Reference	
Male	1.79 (1.67–1.91)	<0.001	1.42 (1.36–1.48)	<0.001
Grade				
Middle school	1.11 (1.03–1.19)	0.003	1.50 (1.44–1.56)	<0.001
High school	Reference		Reference	
Subjective economic state				
High	1.32 (1.22–1.43)	<0.001	1.15 (1.11–1.20)	<0.001
Middle	Reference		Reference	
Low	1.79 (1.65–1.95)	<0.001	1.22 (1.17–1.28)	<0.001
Academic achievement				
High	Reference		Reference	
Middle	1.07 (0.98–1.18)	0.133	0.97 (0.92–1.01)	0.168
Low	1.83 (1.70–1.98)	<0.001	1.00 (0.96–1.05)	0.860
Breakfast consumption				
<2 times/week	1.80 (1.67–1.93)	<0.001	1.05 (1.00–1.09)	0.033
≥2 times/week	Reference		Reference	
Current drinking				
No	Reference		Reference	
Yes	2.33 (2.18–2.49)	<0.001	1.36 (1.30–1.43)	<0.001
Current smoking				
No	Reference		Reference	
Yes	3.65 (3.37–3.94)	<0.001	1.46 (1.37–1.55)	<0.001
Subjective healthiness				
Healthy	Reference		Reference	
Average	1.21 (1.12–1.31)	<0.001	1.06 (1.01–1.10)	0.009
Unhealthy	2.07 (1.87–2.28)	<0.001	1.34 (1.27–1.42)	<0.001
Subjective happiness				
Happy	Reference		Reference	
Average	1.19 (1.10–1.28)	<0.001	1.41 (1.35–1.47)	<0.001
Unhappy	1.84 (1.69–2.01)	<0.001	3.43 (3.25–3.62)	<0.001
Perceived stress				
High	1.25 (1.13–1.38)	<0.001	4.11 (2.86–4.39)	<0.001
Average	0.87 (0.78–0.97)	0.010	1.47 (1.35–1.59)	<0.001
Low	Reference		Reference	
Sleep satisfaction				
Enough	Reference		Reference	
Average	1.07 (1.04–1.89)	<0.001	1.96 (1.12–2.18)	<0.001
Not enough	2.37 (2.17–3.16)	<0.001	6.12 (4.61–7.95)	<0.001
Depression				
No	Reference		Reference	
Yes	2.14 (2.00–2.29)	<0.001	5.44 (5.23–5.67)	<0.001
Suicidal ideation				
No	Reference		Reference	
Yes	2.05 (1.91–2.21)	<0.001	3.12 (1.93–4.13)	<0.001
Suicide attempt				
No	Reference		Reference	
Yes	3.99 (3.61–4.40)	<0.001	2.30 (2.04–2.60)	<0.001

Following the selection of significant covariates, univariate and multivariate logistic regression analyses were performed to identify the associated factors for school absenteeism in adolescents with atopic dermatitis. This yielded odds ratios (OR), adjusted odds ratios (aORs), and 95% confidence intervals (CIs).

## Data Availability

Availability of data and material raw data are available on the official website of the KYRBS, https://www.cdc.go.kr/yhs/home.jsp accessed on 9 October 2020.
